# WHAT DOES LONG-DISTANCE CAREGIVING LOOK LIKE?

**DOI:** 10.1093/geroni/igz038.2056

**Published:** 2019-11-08

**Authors:** Amy Horowitz, Danielle Jimenez, Verena Cimarolli, Francesca Falzarano, Jillian Minahan

**Affiliations:** 1 Fordham University, New York, New York, United States; 2 LeadingAge LTSS Center @UMass Boston, Washington, District of Columbia, United States

## Abstract

Long-distance caregivers (LDCs) are defined by geography, with little known about what they actually do when visiting and from afar. Both quantitative and qualitative data were collected from 304 LDCs. Half of LDCs lived more than 500 miles away from the care receiver (CR); 38% visited at least 1x a month, another 53% visited several times a year. Visit length varied extensively, ranging from one to 90 days at a time, with a median of 3 days. A wide range of care management tasks were common both when visiting and from afar; and targeted both formal providers and other informal caregivers. Emotional support and help with ADLs and IADLs were common during in-person visits. Other examples of emerging themes include: building relationships with formal care providers; personalizing care through, for example, special foods and/or activities; and the role of resources in determining visit length and help provided.

